# Nanoparticle-Doped Hybrid Polyelectrolyte Microcapsules with Controlled Photoluminescence for Potential Bioimaging Applications

**DOI:** 10.3390/polym13234076

**Published:** 2021-11-24

**Authors:** Galina Nifontova, Victor Krivenkov, Mariya Zvaigzne, Anton Efimov, Evgeny Korostylev, Sergei Zarubin, Alexander Karaulov, Igor Nabiev, Alyona Sukhanova

**Affiliations:** 1 Laboratoire de Recherche en Nanosciences, LRN-EA4682, Université de Reims Champagne-Ardenne, 51100 Reims, France; galina.nifontova@univ-reims.fr; 2 Laboratory of Nano-Bioengineering, National Research Nuclear University MEPhI (Moscow Engineering Physics Institute), 115409 Moscow, Russia; victor.krivenkov@ehu.eus (V.K.); mariazvaigzne@gmail.com (M.Z.); 3 Centro de Física de Materiales (MPC, CSIC-UPV/EHU), University of Basque Country (UPV-EHU), Paseo Manuel de Lardizabal 5, 20018 Donostia-San Sebastian, Spain; 4 Shumakov National Medical Research Center of Transplantology and Artificial Organs, 123182 Moscow, Russia; antefimov@gmail.com; 5 Moscow Institute of Physics and Technology (State University), Dolgoprudny, 141701 Moscow, Russia; korostylev.ev@phystech.edu (E.K.); zarubin.ss@mipt.ru (S.Z.); 6 Department of Clinical Immunology and Allergology, Institute of Molecular Medicine, Sechenov First Moscow State Medical University (Sechenov University), 119146 Moscow, Russia; drkaraulov@mail.ru

**Keywords:** quantum dots, magnetic nanoparticles, hybrid polyelectrolyte microcapsules, nanoparticle-encoding, photobrightening, ultramicrostructure

## Abstract

Fluorescent imaging is widely used in the diagnosis and tracking of the distribution, interaction, and transformation processes at molecular, cellular, and tissue levels. To be detectable, delivery systems should exhibit a strong and bright fluorescence. Quantum dots (QDs) are highly photostable fluorescent semiconductor nanocrystals with wide absorption spectra and narrow, size-tunable emission spectra, which make them suitable fluorescent nanolabels to be embedded into microparticles used as bioimaging and theranostic agents. The layer-by-layer deposition approach allows the entrapping of QDs, resulting in bright fluorescent microcapsules with tunable surface charge, size, rigidity, and functional properties. Here, we report on the engineering and validation of the structural and photoluminescent characteristics of nanoparticle-doped hybrid microcapsules assembled by the deposition of alternating oppositely charged polyelectrolytes, water-soluble PEGylated core/shell QDs with a cadmium selenide core and a zinc sulfide shell (CdSe/ZnS), and carboxylated magnetic nanoparticles (MNPs) onto calcium carbonate microtemplates. The results demonstrate the efficiency of the layer-by-layer approach to designing QD-, MNP-doped microcapsules with controlled photoluminescence properties, and pave the way for the further development of next-generation bioimaging agents based on hybrid materials for continuous fluorescence imaging.

## 1. Introduction

Fluorescence sensing and imaging techniques have been actively employed for bioimaging applications, including controlling intracellular acidification, vesicular trafficking, and cytoskeletal reorganizations, as well as in vivo monitoring of delivery, release, and biodistribution [1,2,3]. Dual doping of micro- and nanoparticles with fluorescent and magnetic components has recently been established and frequently used in the design of advanced bioimaging and theranostic tools. A combination of fluorescent organic dyes, or their conjugates, with proteins, polymers, and magnetic nanoparticles (MNPs) is well known to be used in the development of stimulus-responsive diagnostic and therapeutic agents [3,4]. However, the in vivo release of Cy7-labeled BSA used to dope microcapsules has been shown to be initially quenched by MNPs, which was confirmed by fluorescent tomography [2]. In addition, the use of the magnetic component ensures the enhancement of contrast for the magnetic resonance imaging of tumors in vivo [5]. The presence of MNPs also enables the controlled transportation and delivery of the inner content of the microcapsules to the desired location [6]. The simultaneous entrapment of MNPs and fluorescent nanolabels, such as quantum dots (QDs), into polyelectrolyte polymer microcapsules enables their dual sensitivity to light and magnetic fields [7]. The dual doping with light- and magneto-sensitive components makes it possible to enhance the biomedical potential of these systems, enabling their magnetic-field-stimulated transportation, as well as fluorescent and magnetic resonance imaging in the region of interest. The designed polyelectrolyte microcapsules functionalized with metal nanoparticles, drugs, and fluorescent dyes have been recently demonstrated to be effective multifunctional delivery systems that can be easily adapted for theranostic, bioimaging, and sensing applications [8,9,10,11,12,13].

Quantum dots, being fluorescent semiconductor nanocrystals, are characterized by bright, stable fluorescence, a prolonged fluorescence lifetime, and a high quantum yield compared to those of conventional organic dyes and, thus, represent an excellent alternative as fluorescent nanoprobes [14,15]. Core/shell QDs have emerged as excellent fluorescent nanolabels with quantum yield values that may reach 100% [16]. QDs of this type typically represent Cd-based cores (e.g., CdSe, CdTe, or CdS) coated with protective layers of a ZnS or CdS shell to improve the photoluminescence properties of the QD cores, their stability, and their surface functionalization capacity [17,18,19,20]. Core/shell QDs have been successfully introduced as nanolabels for fluorescent imaging, including laser scanning confocal microscopy, super-resolution imaging, and stimulated emission depletion nanoscopy [21,22,23]. However, oxidation of CdZe/ZnS nanocrystals may lead to the leakage of free metal ions from their cores and the resultant toxicity [24]. Core/shell Cd-based QDs possess redox capacity and, along with graphene-based QDs, have also been shown to generate reactive oxygen species (ROSs) [25,26]. Thus, QD encapsulation within the polyelectrolyte shell of the microcapsules prevents direct contact of cells and nanoparticles, which makes it possible to decrease their potential toxicity and enhance their biocompatibility upon their interaction with live cells [27]. Polyelectrolyte microcapsule functionalization with metal nanoparticles, fluorescent dyes, and QDs can be performed using the layer-by-layer approach, which is widely used for microcapsule fabrication [28,29]. QD encapsulation into the polyelectrolyte shell of the microcapsules results in the formation of brightly fluorescent hybrid nanoparticle–polyelectrolyte microstructures that have been demonstrated to be advanced and biocompatible bioimaging tools [30,31].

Layer-by-layer deposition allows for the dual doping of the polyelectrolyte shell with different nanoparticle types, including MNP and fluorescent semiconductor particles. However, the inner complexity of the microcapsule polyelectrolyte shell microenvironment, as well as microcapsule surface charge, has been determined to affect QD fluorescence stability under prolonged laser irradiation, leading to photodarkening or photobrightening of the encapsulated QDs [28].

Our previous research has demonstrated the most stable fluorescence of the QD- and MNP-doped microcapsules that had a negatively charged surface, which acted as a barrier that blocked the transfer of the negative carriers from the QD cores, leading to fluorescence stabilization [28]. However, the poor reproducibility of the layer-by-layer assembly of polyelectrolytes and nanoparticles still represents a challenge in microcapsule engineering [32], demonstrating the necessity to control and validate the structure and optical characteristics of the nanoparticle-doped microcapsules. This study was aimed at the characterization and validation of the structural and fluorescent properties of newly engineered magneto-optical hybrid polyelectrolyte microcapsules dually doped with QDs and MNPs. Our data demonstrate that the designed microcapsules exhibit reproducible and improved fluorescent properties, enabling fluorescence signal detection under continuous irradiation in multicomponent cell culture media, and are promising bioimaging tools for continuous fluorescence imaging.

## 2. Materials and Methods

### 2.1. Fabrication of Nanoparticle-Doped Hybrid Polyelectrolyte Microcapsules

The nanoparticle-doped polyelectrolyte microcapsules were prepared by the electrostatically driven co-assembly of polyelectrolyte polymers on the surface of 5 μm calcium carbonate microparticles used as template cores. The calcium carbonate templates were fabricated via crystallization by mixing equal volumes of equimolar solutions of calcium chloride and sodium carbonate as described elsewhere [30,33]. Initially, 0.5 mL of a suspension containing ~10^8^–10^9^ calcium carbonate microparticles was prepared and sonicated using an ultrasound bath. Then, 0.5 mL of a 2 mg/mL poly (allylamine) hydrochloride (PAH) solution (Mw = 65 kDa, Merck Group, Sigma-Aldrich, Saint-Quentin-Fallavier, France) in 0.5 M sodium chloride was added to 0.5 mL of the suspension and incubated for 20 min under permanent stirring. After incubation, the polymer solution was replaced with ultrapure water by centrifugation, and the resultant pellet was washed thrice. Then, a similar procedure was performed to apply poly (sodium 4-styrenesulfonate) (PSS, Mw = 70 kDa, Merck Group, Sigma-Aldrich, Saint-Quentin-Fallavier, France). A 2 mg/mL PSS solution in 0.5 M sodium chloride was used. The alternating cycles of the deposition of PAH and PSS polyelectrolytes were repeated until the desired shell thickness was achieved. The polyelectrolyte shell was assembled and simultaneously functionalized with both types of nanoparticles using the layer-by-layer approach as described earlier [28,34].

For this purpose, water-soluble core/shell CdSe/ZnS QDs with a fluorescence maximum at 594 nm solubilized with a polyethyleneglycol (PEG) derivative containing a 12-unit PEG-spacer arm and thiol and carboxylic functional groups were prepared as described elsewhere [8,30,35]. Carboxylated iron (II, III) oxide MNPs (Merck Group, Sigma-Aldrich, Saint-Quentin-Fallavier, France) were used for polyelectrolyte shell functionalization. The microcapsules were labeled with the prepared solubilized QDs via adsorption onto the calcium carbonate microparticles that were preliminarily coated with the (PAH/PSS)_2_/PAH layers as described elsewhere [28]. After QD layer deposition, the PAH/PSS/PAH or (PAH/PSS)_2_PAH polyelectrolyte sequence was assembled for further MNP adsorption. Finally, the preformed MNP layer was coated with (PAH/PSS)_3_. Negatively charged microcapsules were prepared by applying the final layer of polyacrylic acid (Mw = 100 kDa, Merck Group, Sigma-Aldrich, Saint-Quentin-Fallavier, France) to obtain the (PAH/PSS)_2_/PAH/PAA sequence. Hollow microcapsules were produced by the incubation of the suspended microparticles in EDTA solution.

### 2.2. Measurements of the Zeta-Potential and Size

The charge and hydrodynamic diameter distribution analyses were performed by means of laser Doppler electrophoresis and dynamic light scattering using a Zetasizer Nano-ZS instrument (Malvern Panalytical, Palaiseau, France). Additionally, the microcapsule size was verified by optical microscopy.

### 2.3. Analysis of Microcapsule Photoluminescence

The photoluminescence spectra of the QD-, MNP-encoded hybrid microcapsules were analyzed using an Infinite 200 PRO multimodal plate reader (TECAN, Männedorf, Switzerland).

The photoluminescence signal stability of the QD-, MNP-doped microcapsules was investigated by the continuous irradiation of the microcapsule suspension containing 6×10^6^ particles using a homemade setup [28,36]. The microcapsule sample was irradiated under permanent stirring. The laser radiation power was detected using a Nova II (Ophir) power meter. The PL intensity of the sample during irradiation was measured using a two-lens objective to collect the light emitted by the sample to an M266 monochromator spectrograph (Solar Laser Systems) with a connected Hamamatsu photodetector matrix.

Microcapsule fluorescence was also investigated using fluorescence microscopy and microcapsule sections as described elsewhere [28]. Fluorescent images were obtained using an Axio Vert.A1 fluorescent microscope (Carl Zeiss, Jena, Germany). The images were analyzed and processed using Zen software (Carl Zeiss).

### 2.4. Transmission Electron Microscopy

The 70 nm-thick microcapsule sections were prepared as described in detail elsewhere, placed onto 200 mesh Formvar/carbon grids (Agar Scientific, Essex, UK), and analyzed using a JEM-2100 transmission electron microscope (JEOL, Akishima, Japan) as described elsewhere [8,28].

### 2.5. Preparation of Microcapsule Sections and Study of Magnetization Distribution

The surface of the microcapsule section was analyzed using atomic force microscopy (AFM) and magnetic force microscopy (MFM) by means of a combined SPM–ultramicrotomy system. The microcapsule section surface was initially scanned in the AFM semicontact mode; then, a phase image indicating the distribution of magnetic forces at the microcapsule section was acquired using MFM_HC magnetic cantilevers (Tipsnano OÜ, Tallinn, Estonia) with probes covered with CoCr (resonance frequency, 64.2 kHz) for magnetization distribution analysis as described earlier [28,37].

## 3. Results and Discussion

### 3.1. Engineering of Nanoparticle-Doped Hybrid Polyelectrolyte Microcapsules

**Figure 1:**
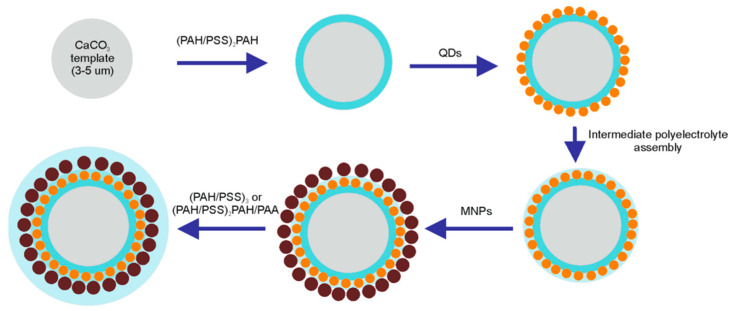
Schematic diagram of nanoparticle deposition during the assembly of the polyelectrolyte shell of the microcapsules. Abbreviations: QD, quantum dot; MNP, magnetic nanoparticle; PAH, poly (allylamine) hydrochloride; and PSS, poly (sodium 4-styrene sulfonate).

Layer-by-layer adsorption of polyelectrolytes has been shown to be a promising approach to the encapsulation of aqueous nanoparticle colloids, which enables further stimulus-responsiveness of the polyelectrolyte shell to light and/or magnetic field stimuli. Dual microcapsule functionalization can be performed during polyelectrolyte shell formation due to electrostatically driven adsorption onto calcium carbonate microbeads previously coated with polyelectrolyte layers (Figure 1). The efficiency of the layer-by-layer deposition of polyelectrolytes and nanoparticles is determined by the surface properties of the templates, charge of polyelectrolyte molecules, zeta-potential, and colloidal stability of the nanoparticles to be encapsulated in the preformed polyelectrolyte shell.

The first step in the fabrication of the microcapsules involves the validation of the charge characteristics of the major building blocks (templates, polyelectrolytes, and nanoparticles). The charge characteristics of the microcapsule building blocks used for the engineering of nanoparticle-doped hybrid microcapsules are presented in Table 1. Therefore, controlling the surface charge during the deposition of the major functional components is a crucial step in the validation of the microcapsule fabrication approach. Our earlier data indicated flips of the microcapsule surface charge upon subsequent deposition of polyelectrolytes and nanoparticles [8,28,30].

**Figure 2:**
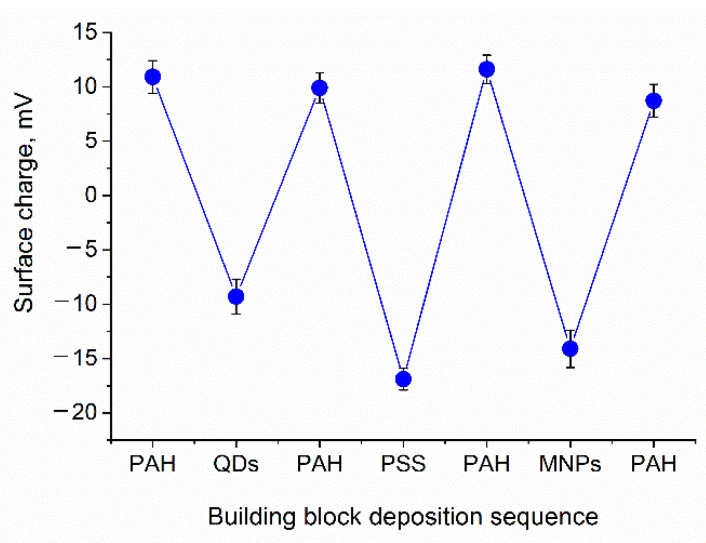
Flips of the surface charge of the calcium carbonate microbead templates (CaCO_3_/(PAH/PSS)_2_) during the deposition of nanoparticles and the intermediate polyelectrolyte layers PAH/PSS/PAH. Abbreviations: QD, quantum dot; MNP, magnetic nanoparticle; PAH, poly (allylamine) hydrochloride; and PSS, poly (sodium 4-styrene sulfonate). Error bars indicate the standard deviations of the mean values of the surface charges measured (the number of measurements was 5).

Nanoparticles (QDs as well as MNPs) were assembled onto the surface of the PAH-coated calcium carbonate microbeads. Both QDs and MNPs bore carboxylic functional groups on their surface and had a negative surface charge, which enabled effective electrostatically driven nanoparticle adsorption onto positively charged, PAH-coated microbeads. After the QDs were applied intermediate alternating PAH/PSS polyelectrolyte layers of the desired total thickness were assembled. Then, MNPs were absorbed from the colloidal solution onto the calcium carbonate microbead surface with pre-assembled QD–polyelectrolyte layers terminated with PAH; then, the surface was coated with a final succession of polyelectrolyte layers resulting in the formation of the following structures: CaCO_3_/(PAH/PSS)_2_/PAH/QDs/PAH/PSS/PAH/MNPs/(PAH/PSS)_3_ or CaCO_3_/(PAH/PSS)_2_/PAH/QDs/PAH/PSS/PAH/MNPs/(PAH/PSS)_2_/PAH/PAA. The functionalization of the polyelectrolyte shell with nanoparticles was characterized by flips of the particle surface charge (Figure 2). Nanoparticle sorption from the colloidal solution was accompanied by overcharging of the template surface, indicating the deposition of negatively charged QDs and MNPs.

**Figure 3:**
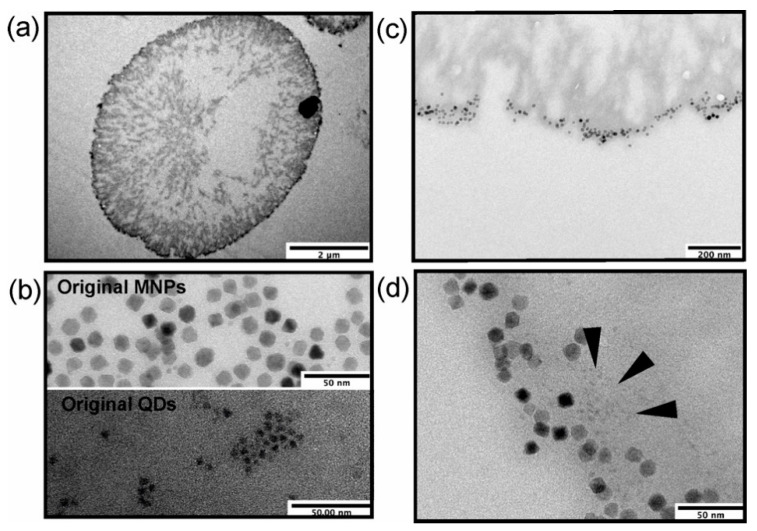
Patterns of nanoparticle deposition in the microcapsule shell as determined by transmission electron microscopy (TEM): (**a**) a section of a nanoparticle-doped hybrid polyelectrolyte microcapsule; (**b**) original MNPs and QDs used for microcapsule functionalization (scale bars, 50 nm); (**c**,**d**) selected regions of the microcapsule shell doped both with QDs and MNPs at different magnifications (scale bars, 200 and 500 nm, respectively). The deposition area of some QDs is indicated with dark arrows. The data on (PAH/PSS)_2_/PAH/QDs/PAH/PSS/PAH/MNPs/(PAH/PSS)_3_ nanoparticle-doped microcapsules are presented. Abbreviations: QDs, quantum dots; MNPs, magnetic nanoparticles; PAH, poly (allylamine) hydrochloride; and PSS, poly (sodium 4-styrene sulfonate).

To estimate the efficiency of the layer-by-layer deposition of QDs and MNPs, a transmission electron microscopy (TEM) analysis of microcapsule sections was performed. The results of microparticle encoding with QDs and MNPs were estimated using TEM analysis (Figure 3). The deposition of nanoparticles into the shell was observed, with nanoparticles of both types forming multilayered assemblies (Figure 3a,c,d), which was especially typical of QDs as smaller-sized particles (Figure 3b,d). The MNPs embedded in the microcapsules had a larger physical size (Figure 3b) and were also assembled in non-uniform layers (Figure 3c,d). In addition, the rough surface of the polyelectrolyte-coated calcium carbonate microbeads (Figure 3a) may also have contributed to the nanoparticle deposition pattern [30].

**Figure 4:**
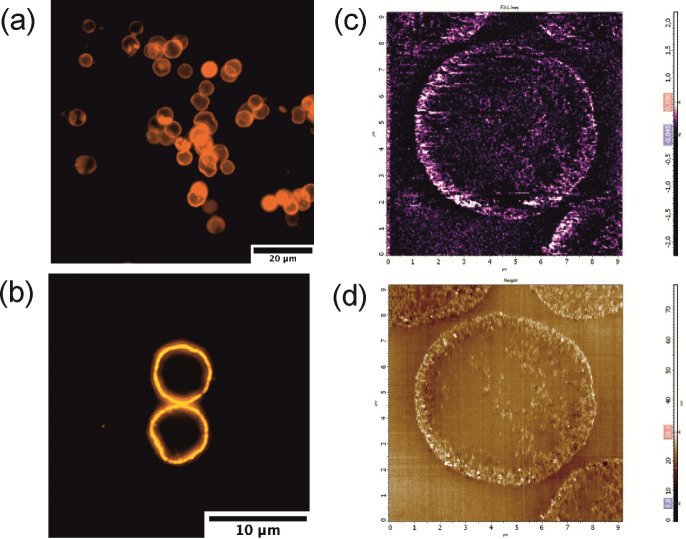
Fluorescence and magneto-responsive properties of the QD-, MNP-doped hybrid polyelectrolyte microcapsules: (**a**) an overview image of fluorescent microcapsules; (**b**) an image of a selected microcapsule section; (**c**) magnetic force microscopy and (**d**) atomic force microscopy images of a single microcapsule section; scan size, 10.9 μm × 10.9 μm; scanning was performed at a rate of 0.85 Hz.

The QD deposition pattern was also verified using a fluorescence microscopy analysis. The uniform fluorescence of the microcapsule shell is shown in Figure 4. The obtained data showed homogeneous distribution of the fluorescent nanoparticles in the polyelectrolyte shell (Figure 4a,b), which agrees with the transmission electron microscopy (TEM) results.

Additionally, to verify the MNP localization in the microcapsule shell, magnetic force microscopy (MFM) of the microcapsules dually doped with both QDs and MNPs was performed. The areas of the highest surface magnetization lying around the microcapsule shell correspond to the MNP deposition sites (Figure 4c). Results of MFM measurements of the microcapsule sections were controlled by AFM topography scanning (Figure 4d).

### 3.2. Analysis of Microcapsules’ Photoluminescence Properties

The embedment of the QDs within the polyelectrolyte shell of the microcapsules could lead to an alternation of their fluorescence properties; in particular, a slight red shift (up to 2 nm) of the fluorescence maximum of the CdSe/ZnS QDs was observed earlier after immobilization between polycation layers [30,35] Therefore, first, the fluorescence spectra of the designed QD-, MNP-encoded microcapsules were estimated. The resultant microcapsules were characterized by fluorescence maxima close to those of the original QDs used for microcapsule encoding, and the red shift did not exceed 2 nm (Figure S1).

To estimate the PL signal stability of the QDs embedded within the interpolymer matrix of the microcapsule shell, both positively and negatively charged PSS microcapsules dually doped with both nanoparticle types were prepared and continuously irradiated with a laser. The PL properties of the engineered QD-, MNP-doped hybrid microcapsules were investigated under prolonged irradiation in a multicomponent cell culture media widely used for cell culturing and, therefore, actively employed in live-cell imaging studies, where salted buffer solutions are unsuitable for long-term cell maintenance [38,39]. However, most cell culture media contains phenol red, and thus exhibit fluorescence. Although background fluorescence of the standard phenol-red-containing media can be cut off during confocal measurements using an optimized filter set up, it may nevertheless directly affect the PL signal of the designed QD-, MNP-doped hybrid microcapsules.

**Figure 5:**
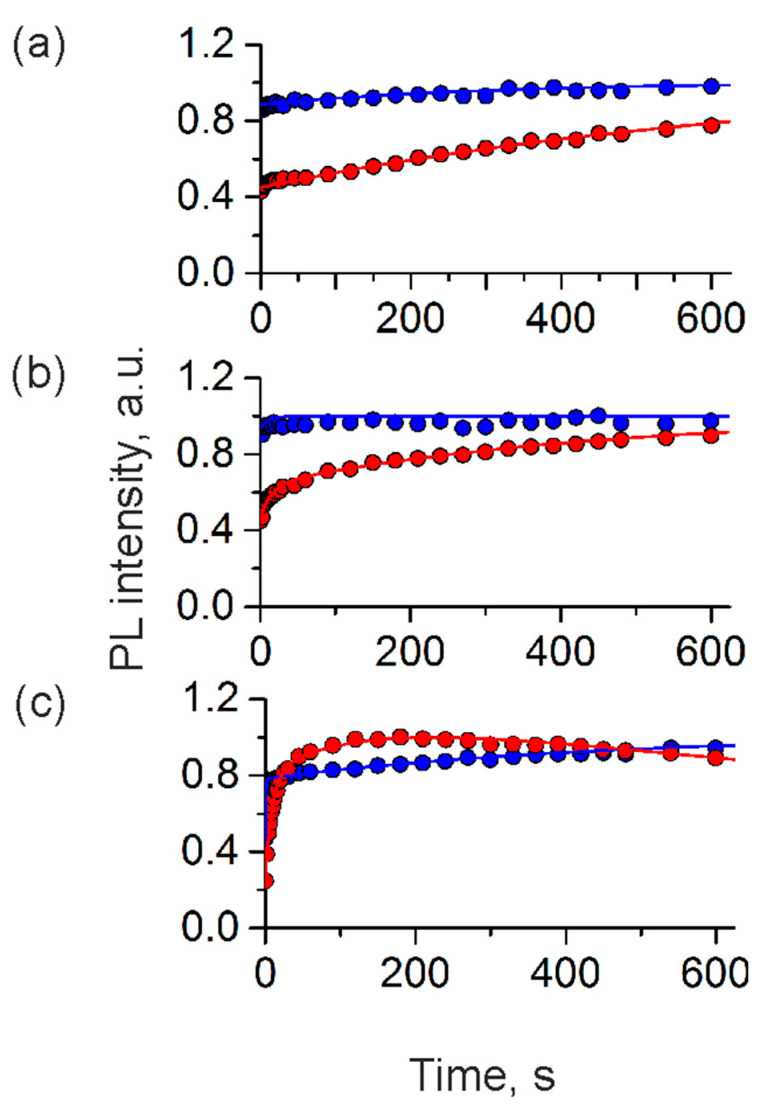
Photoluminescence (PL) kinetics of the QD-, MNP-doped polyelectrolyte microcapsules with negatively and positively charged surfaces in (**a**) the standard phenol-red-containing cell culture medium, (**b**) a phenol-red-free cell culture medium, and (**c**) ultrapure water.

Thus, in this study, we have analyzed the stability of the PL signal of the QD-, MNP-doped microcapsules placed in both the medium containing phenol red and its phenol-red-free modification (Figure 5). Negatively charged PAA-coated microcapsules suspended in the phenol-red-containing medium were characterized by quick initial photobrightening followed by the stabilization of the PL signal, which agrees with our earlier data [28] (Figure 5a). The surface charge of the PAA-coated microcapsules suspended in this medium remained negative and was determined to be −18.2 ± 0.3 mV. In the case of the PSS-terminated microcapsules, which were earlier found to be positively charged (+8.9 ± 0.2 mV), we observed photobrightening kinetics similar to that of the negatively charged samples (Figure 5a). However, the PL stabilization was characterized with a slower rate than in the case of negatively charged microcapsules and took several hundreds of seconds before reaching a permanent PL signal. The zeta-potential measurements showed that there was a flip of the surface charge of the PSS-coated microcapsules in the cell culture medium after irradiation, and the microcapsule surface charge was found to be slightly negative (−7.8 ± 0.5 mV). The observed change in microcapsule surface charge might have led to the deceleration of the migration of the negative charges from QDs to the polyelectrolyte layers according to the model suggested [28].

The photoinduced changes in the photoluminescence of the prepared microcapsules in the phenol-red-free medium were characterized by trends similar to that observed for both negatively and positively charged samples in the standard cell culture medium (Figure 5b). In the case of the positively charged microcapsule samples dispersed in the phenol-red-free medium, a similar flip of the particle surface charge was also detected. The particle surface charge was found to change from positive to negative (−7.2 ± 0.6 mV). The observed changes in the surface charge of the microcapsules suspended in cell culture media can be explained by surface sorption of its negatively charged components (e.g., amino acids) that possibly form an external barrier of negative charges, preventing charge transfer from the QD cores. The control samples of positively and negatively charged microcapsules placed in ultrapure water exhibited the photobrightening kinetics observed earlier [28]. Specifically, positively charged microcapsules exhibited initial photobrightening followed by photodarkening, whereas negatively charged samples exhibited ultrafast initial photobrightening and stabilization of the photoluminescence signal (Figure 5c). The bi-exponential approximation of the results (Equation (1)) of the photoinduced changes in the photoluminescence of polyelectrolyte microcapsules with opposite surface charges in different cell culture media is shown in Table 2.

(1) $$PL=A_{1}e^{-k_{1}t}+A_{2}e^{-k_{2}t}$$

We have previously studied QD-encoded polyelectrolyte microcapsules bearing a negative surface charge as ultrabright tools providing effective fluorescence imaging of their interaction with live phagocytic and cancer cells [30,40]. It has been shown that microcapsule internalization and uptake are driven by a complex mechanism, primarily including the attachment of microcapsules to the cell surface due to strong electrostatic interactions and the subsequent lipid-raft-mediated micropinocytosis of the capsules, which have been established for breast cancer cells. After entering the cytosol, the microcapsules are known to reach heterophagolysosomes, which are considered to be their final localization in cells [41]. The same mechanism has also been reported for normal bone-marrow-derived dendritic cells [42]. However, in the case of normal human vascular smooth muscle cells, it was shown that micropinocytosis, caveola-mediated endocytosis, and cytoskeleton rearrangement took place upon the interaction of these cells with microcapsules. After being internalized by muscle cells, the capsules were found to eventually accumulate around the cell nuclei [43]. Upon interaction with live cells, QD-doped and MNP-doped polyelectrolyte microcapsules did not exhibit significant cytotoxicity, which resulted in a total cell viability of 80–90% in the cases of both normal and cancer cells [27,31,43,44]. These data confirm the efficacy of the encapsulation approach for enhancing the nanoparticle biocompatibility and demonstrate that nanoparticle-doped polyelectrolyte microcapsules can be used as agents to follow particle–cell interaction.

The possibility of ROS generation by MNPs, QDs, or carbon nanotubes significantly restricts their biomedical application [45,46]. However, the encapsulation of MNPs and QDs within the polyelectrolyte has been shown to enhance nanoparticle biocompatibility, probably due to the limitation of direct contact of the nanoparticles with live cells due to their entrapment between polymer layers, which mitigates the possible effect of ROS entities on live cells [27].

## 4. Conclusions

Thus, the obtained results demonstrate the efficacy of the use of water-soluble QDs and MNPs, as well as the layer-by-layer approach to the functionalization of the polyelectrolyte microcapsules. The characterization of the fluorescent and structural properties of the nanoparticle-doped hybrid microcapsules represents a crucial step in the validation of their applicability as agents for prolonged fluorescence bioimaging. The presented data indicate the capacity of the negatively charged, PAA-coated hybrid microcapsule to provide a stable photoluminescence signal, whereas the originally positively charged microcapsules in the cell culture media tested exhibited a delayed stabilization of the optical signal of the QDs encapsulated in the polyelectrolyte shell as the surface charge declines. The obtained data will allow for the further sophistication of the functionality of imaging tools based on QD-encoded microcapsules. The designed QD-, MNP-doped hybrid polyelectrolyte microcapsules are promising stimulus-controlled agents to be used as tools for continuous fluorescence imaging.

## Figures and Tables

**Table 1:** Mean hydrodynamic diameters and ς-potential values of the major microcapsule building blocks.

Building Block	ς-Potential, mV	Hydrodynamic Diameter, nm *
Calcium carbonate microbeads	−8.86 ± 1.14	4137.00 ± 883.70
PSS 70 kDa	−18.10 ± 7.86	309.00 ± 88.03
PAH 65 kDa	+25.0 ± 1.85	320.60 ± 36.13
PAA 100 kDa	−9.30 ± 2.96	298.60 ± 36.90
QDs carboxylated	−36.90 ± 0.31	58.70 ± 9.54
MNPs carboxylated	−41.80 ± 4.24	31.22 ± 0.46

* The data presented by light scattering intensity.

**Table 2:** Approximation results of the photoinduced changes in the photoluminescence of polyelectrolyte microcapsules with opposite surface charges in multicomponent media.

Parameter	Water		Regular Cell Culture Medium		Phenol-Red-Free Cell Culture Medium	
	Positively charged	Negatively charged	Positively charged	Negatively charged	Positively charged	Negatively charged
A_1_	−1.900	−0.100	−0.363	0.065	−1.420	−0.135
k_1_	0.0534	0.063	−0.067	0.096	−0.001	−0.003
A_2_	+2.500	−0.520	−0.807	-	-	-
k_2_	0.001	0.003	−0.002	-	-	-

## Supplementary Materials

The following are available online at https://www.mdpi.com/article/10.3390/polym13234076/s1, Figure S1: Fluorescence spectrum of the nanoparticle-doped hybrid polyelectrolyte microcapsules.
