# Supplementary material for: Nanoparticle-Doped Hybrid Polyelectrolyte Microcapsules with Controlled Photoluminescence for Potential Bioimaging Applications
Source: Polymers (Basel). 2021 Nov 24;13(23):4076. doi: 10.3390/polym13234076 (PMC8658880; doi:10.3390/polym13234076)
Supplement: Supplementary file 1 [file polymers-13-04076-s001.zip › polymers-1463566-supplementary.pdf]

Supplementary Materials

Nanoparticle-Doped Hybrid Polyelectrolyte Microcapsules with Controlled Photoluminescence for Potential Bioimaging Applications

Galina Nifontova [1,2], Victor Krivenkov [2,3], Mariya Zvaigzne [2], Anton Efimov [4], Evgeny Korostylev [5], Sergei Zarubin [5], Alexander Karaulov [6], Igor Nabiev [1,2,6]* and Alyona Sukhanova [1]*

1 Laboratoire de Recherche en Nanosciences, LRN-EA4682, Université de Reims Champagne-Ardenne, 51100 Reims, France
2 Laboratory of Nano-Bioengineering, National Research Nuclear University MEPhI (Moscow Engineering Physics Institute), 115409 Moscow, Russian Federation
3 Centro de Física de Materiales (MPC, CSIC-UPV/EHU), University of Basque Country (UPV-EHU), Paseo Manuel de Lardizabal 5, 20018 Donostia-San Sebastian, Spain
4 Shumakov National Medical Research Center of Transplantology and Artificial Organs, 123182 Moscow, Russian Federation
5 Moscow Institute of Physics and Technology (State University), Dolgoprudny, Moscow Region, 141701 Russian Federation
6 Sechenov First Moscow State Medical University (Sechenov University), 119146 Moscow, Russian Federation

* Correspondence: I.N., igor.nabiev@univ-reims.fr; A. S., alyona.sukhanova@univ-reims.fr

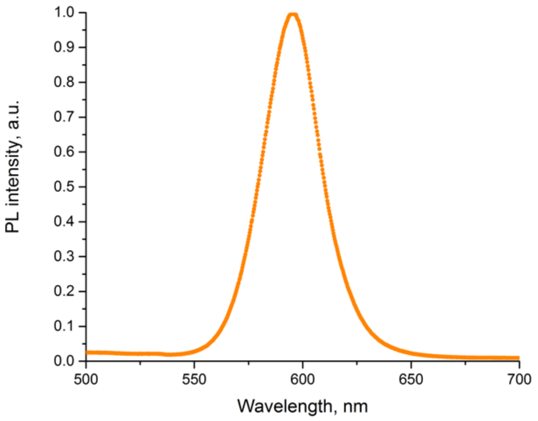

Figure S1. Fluorescence spectrum of the nanoparticle-doped hybrid polyelectrolyte microcapsules.
